# Simplified form of tinnitus retraining therapy in adults: a retrospective study

**DOI:** 10.1186/1472-6815-8-7

**Published:** 2008-11-03

**Authors:** Hashir Aazh, Brian CJ Moore, Brian R Glasberg

**Affiliations:** 1 Audiology Department, Ealing Hospital, Uxbridge Road, London, UB1 3HW, UK; 2 Department of Experimental Psychology, University of Cambridge, Downing Street, Cambridge, CB2 3EB, UK

## Abstract

**Background:**

Since the first description of tinnitus retraining therapy (TRT), clinicians have modified and customised the method of TRT in order to suit their practice and their patients. A simplified form of TRT is used at Ealing Primary Care Trust Audiology Department. Simplified TRT is different from TRT in the type and (shorter) duration of the counseling but is similar to TRT in the application of sound therapy except for patients exhibiting tinnitus with no hearing loss and no decreased sound tolerance (wearable sound generators were not mandatory or recommended here, whereas they are for TRT). The main goal of this retrospective study was to assess the efficacy of simplified TRT.

**Methods:**

Data were collected from a series of 42 consecutive patients who underwent simplified TRT for a period of 3 to 23 months. Perceived tinnitus handicap was measured by the Tinnitus Handicap Inventory (THI) and perceived tinnitus loudness, annoyance and the effect of tinnitus on life were assessed through the Visual Analog Scale (VAS).

**Results:**

The mean THI and VAS scores were significantly decreased after 3 to 23 months of treatment. The mean decline of the THI score was 45 (SD = 22) and the difference between pre- and post-treatment scores was statistically significant. The mean decline of the VAS scores was 1.6 (SD = 2.1) for tinnitus loudness, 3.6 (SD = 2.6) for annoyance, and 3.9 (SD = 2.3) for effect on life. The differences between pre- and post-treatment VAS scores were statistically significant for tinnitus loudness, annoyance, and effect on life. The decline of THI scores was not significantly correlated with age and duration of tinnitus.

**Conclusion:**

The results suggest that benefit may be obtained from a substantially simplified form of TRT.

## Background

Tinnitus retraining therapy (TRT) is aimed at removing negative associations of the tinnitus signal to enable the natural habituation process to occur [1]. The goal is to achieve this through retraining counseling and sound therapy. Retraining counseling is a crucial part of TRT; it teaches patients the components of the neurophysiological model of tinnitus and encourages them to reclassify their tinnitus as a neutral signal. Sound therapy is assumed to facilitate tinnitus habituation by decreasing the strength of tinnitus signal [2]. The TRT protocol requires that the patient adheres to the regimen for 12–24 months (typically attending for seven sessions over that time), except for patients experiencing weak tinnitus, which has little impact on everyday life.

Since the first description of TRT in the 1990s, clinicians have modified and customised the method of TRT to suit their practice and their patients [3-5]. A simplified form of TRT has been used at Ealing Primary Care Trust (PCT) Audiology Department since 2005. This is different from TRT in the type and (shorter) duration of retraining counseling. Although the counseling used in simplified TRT also aims to get the patient to reclassify tinnitus as a neutral stimulus, it is different from the counseling used in TRT in the following ways: (1) there is no teaching about basic functions of the auditory system; (2) there is no presentation of the basics of brain function and the interactions of various systems of the brain; (3) there is no explanation of the theoretical basis of habituation based on the Jastreboff neurophysiological model; and (4) the duration of the initial counseling of simplified TRT is 30 minutes in comparison to 90 minutes for the initial TRT counseling.

Sound therapy for simplified TRT is the same as for the TRT except for patients in Jastreboff's "category one" [2]. Patients in this category have bothersome tinnitus, but no hearing loss, and no decreased sound tolerance (DST). In simplified TRT, they are issued with a bedside/tableside sound generator (SG) but, in contrast to TRT, wearable sound generators (WSG) are not offered unless the patient asks for them (for more details, see the procedures). The entire simplified TRT takes between 3 and 24 months (2–8 sessions). The first appointment lasts about 30 minutes, and then the patient is seen for follow ups (30 minutes) as required at 1 month, 2 month, 3 month, and 6 month intervals.

The aims of this observational study were: (1) to assess the effectiveness of simplified TRT, as carried out at Ealing PCT Audiology Department during 2005 and 2006 and (2) to determine the extent to which the success of simplified TRT is affected by the duration of tinnitus, the patient's age, the use of hearing aids (HAs), and the use of SGs.

## Methods

### Subjects and Sample Size

Data were collected from a series of 42 consecutive patients (23 males and 19 females) who were referred from the ENT department to the tinnitus clinic at Ealing PCT Audiology Department during 2005–2006. The selection criteria were that each patient: (1) completed the self-assessment questionnaires, (2) attended at least two therapy sessions and continued the treatment for at least 3 months, and (3) exhibited mild to severe tinnitus handicap based on the Tinnitus Handicap Inventory (THI) [6] total score prior to treatment (total THI score ≥ 18). These patients were treated according to our tinnitus clinic policies and procedures and were not recruited simply to take part in a trial.

### Procedures

In the assessment session prior to application of the simplified form of TRT, a general medical history was obtained and otoscopy and pure-tone audiometry were performed. Audiometric thresholds were measured in a sound-attenuating room following the British Society of Audiology recommended procedure [7]. Loudness Discomfort Levels (LDLs) were measured at 0.25, 0.5, 1, 2, 4, 6 and 8 kHz, following the protocol described by Jastreboff and Hazell [8]. Decreased sound tolerance (DST) was considered as present when average LDLs were 90 dB HL or lower and the patient complained about the loudness of environmental sounds.

In the first simplified TRT session, all patients received general information and directive counseling on tinnitus. This counseling was based on explanation of the nature of tinnitus and how to manage it. Its aims were: (1) to reassure patients that the annoyance from tinnitus would gradually reduce with the passage of time following the natural process of habituation; (2) to inform them that reduction in annoyance and distress caused by the tinnitus would promote habituation to the tinnitus and reduction of the tinnitus itself; (3) in cases of tinnitus combined with hearing loss to explain that if they could not hear properly, this was most likely because of their hearing loss and not the tinnitus; and (4) to advise them to avoid silence by using sound enrichment [9].

Sound therapy for simplified TRT was almost the same as for TRT. The specific treatment strategy that was applied to patients in the different categories described by Jastreboff [10] (excluding patients in category 0, who did not form part of the study) is detailed below:

(a) Patients with bothersome tinnitus, but no hearing loss, and no DST were advised about sound enrichment, but WSGs were not offered unless requested. This is the way in which the sound therapy for simplified TRT differs from that for TRT; the latter recommends usage of bilateral WSGs for at least 8 hours per day for patients in this category. In simplified TRT, if the patient asked for WSGs, then bilateral WSGs (Viennatone, Silent Star) were fitted with the same procedure as for TRT, using completely open fittings (Oticon Comfort Tips or skeleton open molds). As for TRT, the patient was instructed to set the volume so that both the tinnitus and the noise generated by the device could be heard.

(b) Patients with tinnitus and hearing loss were advised about sound enrichment and were fitted with digital hearing aids (HAs). This was similar to TRT except that patients were not given the option of combination devices (a combination of a HA and a broadband noise generator), whereas this would be an option for TRT.

(c) Patients exhibiting DST, with tinnitus and with or without hearing loss were advised to use bilateral WSGs and instructed to set the volume of the WSGs at a level that avoided discomfort while making the WSG noise audible in the presence of background environmental noises (instructions were to increase the volume in noisy environments). Initially, the therapy was focused on the DST, and after the patient showed improvement in DST, the tinnitus was addressed more directly. This was similar to TRT.

WSGs and HAs were fitted free under the National Health Service, but patients had to buy the SGs from the supplier. It was explained to patients that WSGs and SGs might facilitate tinnitus habituation by decreasing the strength of the tinnitus signal. It was also explained that HAs may help: (1) to reduce the effort of hearing, and (2) to amplify background noises and facilitate tinnitus habituation by decreasing the strength of the tinnitus signal. However, patients needed to decide for themselves whether or not to proceed with sound therapy of any form.

A single specialist (the first author) administered the treatment. He was clinically certified as an audiologist and had special expertise in tinnitus rehabilitation. Each patient was seen at 2–7 clinical appointments over a period of 3–23 months. The follow up appointments were arranged as required at 1 month, 2 month, 3 month, and 6 month intervals. The outcome measurement questionnaires were completed at the beginning of each session. The scores achieved in the last session were compared with the pre-treatment scores. Patients received about 1 to 3.5 hours of counseling. This excludes the assessment session, which usually took about 45 minutes for measurement of pure tone audiometry and LDLs, taking a case history, and obtaining the baseline questionnaires.

This study was a clinical audit approved by the Clinical Governance department at Ealing PCT and it was designed to assess the Ealing PCT Audiology Department performance. This study also was performed in accordance with the Helsinki declaration on medical ethics issues.

### Outcome measures

Two self-report outcome measures were used: the THI and the Visual Analog Scale (VAS) [11] of tinnitus loudness, annoyance and effect on life. The THI has 25 items, and response choices are "no" (0 points), "sometimes" (2 points) and "yes" (4 points). The overall score ranges from 0 to 100. Scores from 0–16 show no handicap (data from patients exhibiting no handicap were excluded from the current report), scores from 18–36 show mild handicap, scores from 38–56 indicate moderate handicap, and scores from 58–100 show severe handicap [6].

VAS scores are ratings on a scale from 0 to 10. The VAS score for loudness of tinnitus was assessed by asking the patient to rate the loudness of tinnitus during their waking hours over the last month (It was explained that 0 corresponds to no tinnitus being heard and 10 is as loud as gunfire). The VAS score for annoyance induced by the tinnitus was assessed by asking the patient to rate their subjective perception of annoyance on average during the last month (It was explained that 0 corresponds to no annoyance and 10 is the most annoying thing which can possibly happen). The VAS score for the impact of tinnitus on their life was assessed by asking the patient to rate the effect of tinnitus on their life during the last month (It was explained that 0 corresponds to no effect and 10 is as big as an earthquake).

## Results

### Participants and severity of tinnitus symptoms

The age of the patients ranged between 28 and 81 years, with a mean of 60 years (SD = 13). None of them had any kind of previous treatment for tinnitus. The average duration of tinnitus was 6.4 years (SD = 7), with a range between 6 months and 30 years. 35 patients had a hearing loss (hearing loss was defined as pure tone average, PTA, for frequencies of 0.5, 1, 2, and 4 kHz more than 20 dB for at least at one ear) and seven patients had normal hearing. Among the cases with hearing loss, eight were HA users and 27 had never had HAs. Three patients exhibited DST. Two patients had tinnitus in the right ear, 11 had tinnitus in the left ear and 29 had tinnitus in both ears. Table 1 shows patients' descriptions of the quality of their tinnitus.

**Table 1:** Patients' descriptions of the quality of their tinnitus

**Quality of tinnitus**	**Number of patients**	**Percentage (approximate)**
Buzzing noise	8	19%
High pitch noise	5	12%
Hissing noise	5	12%
Whistle	5	12%
Waterfall and grinding wheel	4	10%
Ringing	1	2%
White noise	1	2%
Strong wind	1	2%
Airplane taking off	1	2%
Bubbles and clicks	1	2%
Beep	1	2%
Humming noise	1	2%
Not able to describe	8	19%

According to the THI scores prior to treatment, seven patients (16.7%) had mild handicap, 11 patients (26.2%) had moderate handicap, and 24 patients (57.1%) had severe handicap. For the VAS scores, prior to treatment, 36 patients (85%) ranked the loudness of their tinnitus as ≥ 5, 37 patients (88%) ranked the annoyance induced by their tinnitus as ≥ 5 and 31 patients (74%) ranked the impact of tinnitus on their life as ≥ 5.

### Decline in THI and VAS scores after simplified TRT

As shown in table 2, THI and VAS scores for tinnitus loudness, annoyance and effect on life declined after simplified TRT, indicating a decrease in the subjective handicap produced by the tinnitus. The mean decline of THI scores was 45 (SD = 22). The THI score improved for all patients after treatment and 26 (62%) patients exhibited a decline of 40 or more on the THI score. A one-way repeated-measures analysis of variance (ANOVA) showed a significant difference between THI scores before and after treatment: F(1,82) = 173.8, p < 0.001.

**Table 2:** Means and standard deviations (SDs) of the THI and VAS scores before and after 3–23 months of simplified TRT

	**THI**		**VAS** **Loudness**		**VAS** **Annoyance**		**VAS** **Effect on life**	
	***Mean***	***SD***	***Mean***	***SD***	***Mean***	***SD***	***Mean***	***SD***

***Pre***	60.0	18.9	6.2	1.7	6.5	2.0	6.0	2.1
***Post***	15.3	11.2	4.6	1.5	2.9	1.9	2.1	1.6

The mean decline of the VAS score for tinnitus loudness was 1.6 (SD = 2.1). 23 (55%) patients exhibited a decline of two or more points, but four patients showed increased scores following treatment. The mean decline of the VAS score for annoyance of tinnitus was 3.6 (SD = 2.6). 33 (78%) patients exhibited a decline of two or more points, but one patient showed worse scores following treatment. Finally, 36 (85%) patients showed a decline of the effect of tinnitus on life of 2 or more points, but one patient showed worse scores following treatment. The mean decline of the effect of tinnitus on life was 3.9 (SD = 2.3). A two-way repeated-measures ANOVA on the VAS scores with factors before versus after treatment and VAS sub-scale showed a significant effect of treatment, F(1,246) = 173.1, p < 0.001, a significant effect of VAS sub-scale, F(2, 246) = 11.22, p < 0.001, and a significant interaction, F(2, 246) = 10.49, p < 0.001. Post-hoc comparisons, based on Fisher's least-significant differences test, showed that the decline in scores following treatment was significant for all VAS subscales at p < 0.001.

### Effect of SGs, HAs and WSGs on the outcome of simplified TRT

20 out of the 42 patients used SGs as part of their treatment. The main reasons for rejecting SGs were either having severe hearing loss or having no sleep problems. 28 out of 35 patients with hearing loss used HAs. The seven patients who didn't use HAs had only slight hearing loss (the PTA was between 21 and 30 dB in the worse ear), and they did not feel that they needed HAs. Table 3 shows the mean declines and SDs of the THI and VAS scores for patients who did and did not use SGs and HAs.

**Table 3:** Means (SDs in parentheses) of the decline in THI and VAS scores for patients who used and did not use SGs and HAs

	***THI***	***VAS loudness***	***VAS annoyance***	***VAS effect on life***
**Used SG**	55.6 (20)	1.9 (2.1)	3.6 (2.5)	4.5 (2.4)
**Did not use SG**	34.9 (18)	1.2 (2.1)	3.6 (2.7)	3.4 (2.2)
**Used HAs**	45.6 (24)	1.2 (2.0)	3.8 (2.7)	4.2 (2.2)
**Did not use HAs**	43.4 (19)	2.0 (2.5)	3.4 (1.9)	4.1 (2.5)

An ANOVA was conducted on the THI scores with before versus after treatment as a within-subjects factor and use or non-use of SGs as a between-subjects factor. The effect of before versus after treatment was significant, F(1,40) = 228.3, p < 0.001, and the effect of use of SGs was also significant, F(1,40) = 6.97, p = 0.012. However, a similar ANOVA on the VAS scores, with VAS sub-scale as a within-subjects factor, revealed that the use of SGs did not have a significant effect at the 0.05 level: F(1,40) = 3.25, p = 0.079.

An ANOVA was conducted on the THI scores with before versus after treatment as a within-subjects factor and use or non-use of HAs as a between-subjects factor. The effect of before versus after treatment was significant, F(1,40) = 175.6, p < 0.001, but the effect of use of HAs was not significant, F(1,40) = 0.09, p = 0.766. A similar ANOVA on the VAS scores, with VAS sub-scale as a within-subjects factor also showed that the use of HAs did not have a significant effect: F(1,40) = 0.14, p = 0.708. This result does not support the idea that the use of HAs is critical for producing a decrease in tinnitus handicap. However, because all of the seven patients with tinnitus and hearing loss who did not use HAs as a part of their treatment had only slight hearing loss, this result is only applicable to patients with slight hearing loss. Due to the small number of patients in this group (only seven), this conclusion should be interpreted with caution. There is a need for further research using a controlled trial to assess the efficacy of HAs as a part of treatment for patients exhibiting tinnitus combined with slight hearing loss.

Two out of seven patients with normal hearing used bilateral WSGs. These patients particularly asked for some kind of instrumentation which could help them to cope with their tinnitus in the daytime. The remaining five normally hearing patients did not ask for any assistive device and were not offered WSGs. All of the seven patients with normal hearing exhibited a 20 or more point decline in THI scores following simplified TRT treatment. Due to the small number of patients with normal hearing we are unable to draw any conclusions regarding the efficacy of WSGs for this group.

### Relation between decline in THI score and age and duration of tinnitus

**Figure 1:**
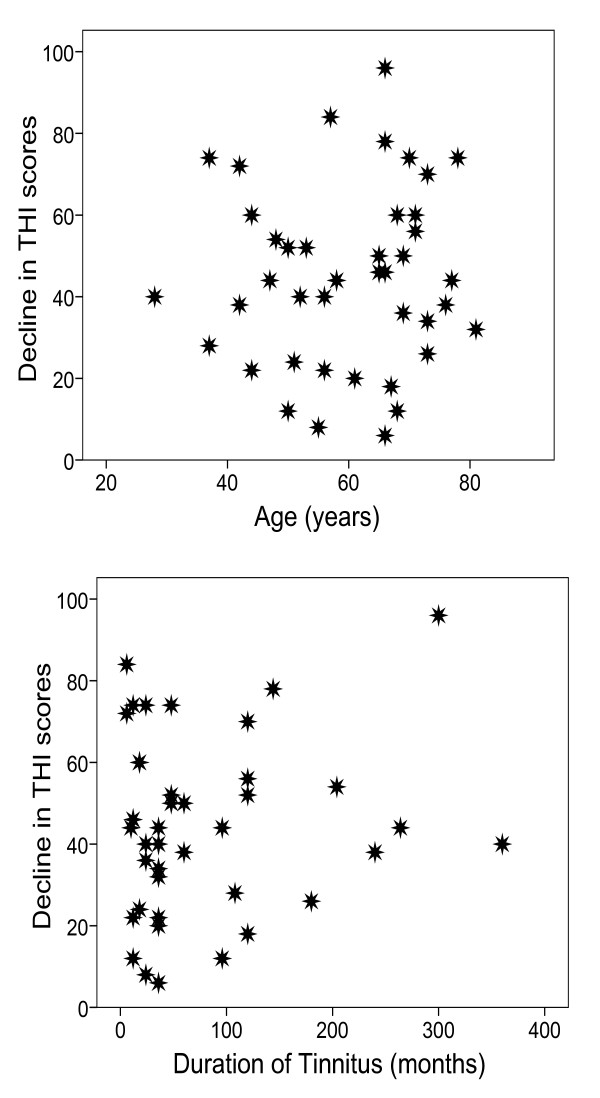
**Scatter plots of the decline of THI scores (improvement in tinnitus handicap) as a function of age (top panel) and duration of tinnitus (bottom panel) for 42 patients who underwent simplified TRT for 3–23 months.** The decline of the THI score is defined as the pre-treatment score minus the post-treatment score for each individual.

As shown in figure 1, the decline in THI scores following simplified TRT did not have a significant linear correlation with age (*r *= 0.063, *p *= 0.69). This suggests that, regardless of the patient's age, from 28 to 81 years, they can receive benefit from simplified TRT. There was no statistically significant linear correlation between the decline in THI scores following treatment and the self-reported length of time the patient had tinnitus (*r *= 0.129, *p *= 0.414). This indicates that, whenever the patient decides to seek professional help for tinnitus, from 5 months to 30 years after the onset of the tinnitus, simplified TRT is capable of providing a substantial reduction in tinnitus handicap.

## Discussion

Educational retraining counseling is generally regarded as an important component of TRT. The counseling in TRT is intended to explain the mechanisms underlying the tinnitus, based on the Jastreboff neurophysiological model, and to remove negative associations with the tinnitus. This is regarded as important for allowing habituation to the tinnitus to occur [2]. The counseling used in Ealing PCT Audiology Department was also intended to reduce negative associations with the tinnitus, but was shorter in duration and simplified. The simplified counseling did not include any teaching about the interactions of various systems of the brain, there was no explanation of the Jastreboff neurophysiological model, and the duration of the initial counseling was only 30 minutes. The sound therapy used with simplified TRT for each patient category was essentially the same as for TRT, except that WSGs were not recommended to patients who exhibited tinnitus with no hearing loss and no DST. However, WSGs were fitted to the patients who showed particular interest in making use of such devices.

TRT is an established method of treating tinnitus patients and typically results in a decline (improvement) in THI scores of 25 to 35 points after 12–24 months of treatment [12,13]. Studies on the psychometric adequacy of the THI questionnaire suggest that a decline in THI score of 20 points or more can be considered as a statistically significant improvement in perceived tinnitus handicap [14]. Our results revealed that the THI score declined by approximately 45 points (SD = 22) after 3–24 months of simplified TRT. The cause of the greater mean effect in our study in comparison with earlier studies of TRT is not clear. It might reflect individual differences in the patients, differences in the way that patients were selected for inclusion in the studies, or individual differences in the clinicians' personality and attitude [15]. In any case, our results indicate that simplified TRT can produce benefits comparable to those produced by TRT.

The current study is limited in the following ways: (1) we did not include a control group to eliminate the placebo effects from attending consultation appointments with a specialist; (2) the sample size was relatively small; (3) within the group of patients there was large variability in symptoms, in the type of instrumentation used (HAs, SGs, WSGs) and in the length of treatment. However, the results still indicate the potential benefit of a substantially simplified form of TRT in reducing tinnitus handicap.

Using SGs as a part of sound therapy has been reported to facilitate tinnitus habituation by decreasing the strength of the tinnitus signal [6]. The majority of tinnitus patients using SGs have been reported to experience an improvement in their sleep [16]. In our study, bedside/tabletop SGs were reported to be helpful by many patients. However, considerable improvement in tinnitus handicap was achieved even for those who did not use SGs. The main factors associated with rejecting SGs were either having severe hearing loss or having no sleep problems whatsoever. The mean decline of THI scores for the patients who used SGs as a part of their treatment was about 20% greater than for those who did not use the SGs.

Surr et al. [17,18] recommended that amplification should be considered in the management of tinnitus and that even patients with limited hearing loss might benefit from HAs. In our study, significant improvement was observed for patients with hearing loss and tinnitus both for those who used HAs and for those who did not. There was no statistically significant difference between the mean decline of the THI and VAS scores between those who used HAs and those who did not. However, for the patients who did not use HAs, the PTA was always between 21 and 30 dB in the worst ear, whereas, the PTA was greater than this for 84% of the patients who used HAs. Thus the two groups were not directly comparable, and we cannot draw a firm conclusion regarding the efficacy of HAs for patients exhibiting tinnitus and slight hearing loss. However, our results do suggest that the use of HAs is not essential for reducing tinnitus handicap in people with slight hearing loss.

It is possible that decisions about fitting HAs for patients with slight hearing loss should depend on the patients' preference. If they are interested in and motivated to wear HAs, then HAs as a part of sound therapy may help them in reducing tinnitus-related problems. On the other hand, if they believe that they do not need HAs, they may benefit from simplified TRT even without amplification. This is consistent with the argument of Henry et al. [19] that patients with marginal hearing loss who are not motivated to wear HAs would be unlikely to benefit from the use of HAs as a treatment for their tinnitus.

## Conclusion

The effectiveness of a substantially simplified version of TRT was assessed through an uncontrolled retrospective study on 42 patients seen at Ealing PCT Audiology Department during the period 2005–2006. Simplified TRT differs from TRT in the type and (shorter) duration of the counseling but is similar to TRT in the application of sound therapy. Although we did not include a control group to assess the extent to which patients would have improved without treatment, our results revealed that simplified TRT was successful in reducing tinnitus handicap. THI and VAS scores for tinnitus loudness, annoyance and effect on life declined (improved) significantly over a period of 3 to 23 months for patients who received simplified TRT. The mean decline of THI score was 45 (SD = 22) and the difference between pre- and post-treatment scores was statistically significant. The mean decline of the VAS score was 1.6 (SD = 2.1) for tinnitus loudness, 3.6 (SD = 2.6) for annoyance, and 3.9 (SD = 2.3) for effect on life. The differences between pre- and post-treatment VAS scores were statistically significant in all cases. The amount of improvement in THI scores tended to be greater for patients who used SGs as a part of their treatment, but was not significantly associated with duration of tinnitus and age.

## Competing interests

The authors declare that they have no competing interests.

## Authors' contributions

HA carried out the simplified TRT, designed the study, performed some of the statistical analyses, and prepared the manuscript. BCJM helped in the interpretation of the data and in writing the manuscript. BRG performed some of the statistical analyses.

## Pre-publication history

The pre-publication history for this paper can be accessed here:


